# Impact of stress on male fertility: role of gonadotropin inhibitory hormone

**DOI:** 10.3389/fendo.2023.1329564

**Published:** 2024-01-08

**Authors:** Adeyemi F. Odetayo, Roland E. Akhigbe, Grace E. Bassey, Moses A. Hamed, Luqman A. Olayaki

**Affiliations:** ^1^ Department of Physiology, Federal University of Health Sciences, Ila Orangun, Nigeria; ^2^ Reproductive Biology and Toxicology Research Laboratory, Oasis of Grace Hospital, Osogbo, Nigeria; ^3^ Department of Physiology, Ladoke Akintola University of Technology, Ogbomoso, Nigeria; ^4^ Department of Physiology, University of Uyo, Uyo, Nigeria; ^5^ Department of Medical Laboratory Science, Afe Babalola University, Ado-Ekiti, Nigeria; ^6^ The Brainwill Laboratories and Biomedical Services, Osogbo, Nigeria; ^7^ Department of Physiology, University of Ilorin, Ilorin, Nigeria

**Keywords:** fertility, reproductive hormones, hypothalamus, pituitary, stress, testosterone

## Abstract

Studies have implicated oxidative stress-sensitive signaling in the pathogenesis of stress-induced male infertility. However, apart from oxidative stress, gonadotropin inhibitory hormone (GnIH) plays a major role. The present study provides a detailed review of the role of GnIH in stress-induced male infertility. Available evidence-based data revealed that GnIH enhances the release of corticosteroids by activating the hypothalamic-pituitary-adrenal axis. GnIH also mediates the inhibition of the conversion of thyroxine (T4) to triiodothyronine (T3) by suppressing the hypothalamic-pituitary-thyroidal axis. In addition, GnIH inhibits gonadotropin-releasing hormone (GnRH), thus suppressing the hypothalamic-pituitary-testicular axis, and by extension testosterone biosynthesis. More so, GnIH inhibits kisspeptin release. These events distort testicular histoarchitecture, impair testicular and adrenal steroidogenesis, lower spermatogenesis, and deteriorate sperm quality and function. In conclusion, GnIH, via multiple mechanisms, plays a key role in stress-induced male infertility. Suppression of GnIH under stressful conditions may thus be a beneficial prophylactic and/or therapeutic strategy.

## Graphical Abstract

Schematic illustration of the impacts of GnIH on male fertility, Gonadotropin inhibitory hormone (GnIH) promotes the release of corticosteroids by activating the hypothalamic-pituitary-adrenal axis. GnIH also inhibits the conversion of thyroxine (T4) to triiodothyronine (T3) by suppressing the hypothalamic-pituitary-thyroidal (HPT) axis. In addition, GnIH inhibits gonadotropin-releasing hormone (GnRH), thus suppressing the hypothalamic-pituitary-gonadal (HPG) axis, therefore suppresses circulating luteinizing hormone (LH), follicle-stimulating hormone (FSH), and testosterone. More so, GnIH inhibits kisspeptin release, thus induces a delay in puberty onset. These series of events is accompanied by GnIH-induced degeneration of germ cells, impairment of spermatogenesis, and deterioration of sperm quality and function.

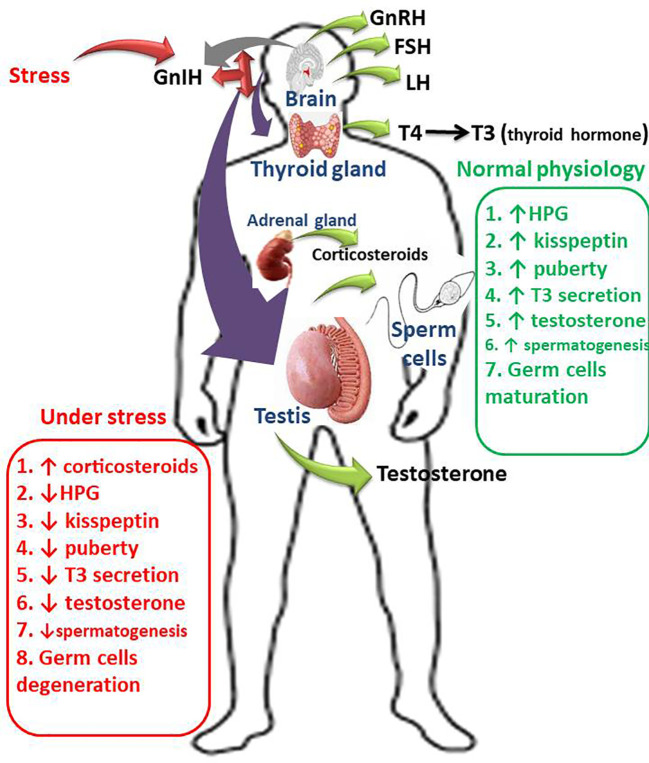

## 1 Introduction

Infertility is a global public health issue that impacts an individual’s social, economic, and personal life (1). According to WHO (2), infertility is a reproductive system disease defined by the inability of clinical pregnancy to be achieved after twelve months of regular unprotected sex. Although infertility does not threaten life, it is depicted as a radical life-changing problem because it bears notable psychological trauma and social stigma (3). Infertility is a very distressful state; the stress associated with it causes a drop in sexual self-esteem, a decrease in the frequency of sexual intercourse, and an increase in marital conflict. Infertile individuals often report feeling less of themselves and inadequate (3). Statistics suggest that an estimated 48.5 million couples worldwide are not fertile, accounting for about 15% of all reproductive couples globally (4). About half of all cases of infertility are contributed by male conditions (5). It is thought that about one-third of cases of being unable to give birth are due to male factors, one-third occur as a result of the female, and the remaining third is due to the combination of male and female factors. In approximately 30% of cases, the cause is labeled as idiopathic, and the condition’s origin is never identified (6). Idiopathic infertility may be explained by the role of mental disorders, such as stress, depression, sleep disorders, eating disorders, and addictions (7).

Stress is any change that disrupts homeostasis by inflicting physical, emotional, or psychological strain (8). Under a stressful condition, the organism modifies its behavior and physiological responses to re-establish homeostasis. Psychological stress caused by a mix of achieving personal targets, hassles, meeting demands, and deadlines, and frustrations is a major type of stress affecting individuals globally (9). In the short-term, stress can motivate and sometimes enhance productivity. The physiologic response to acute stress can also assist in maintaining good health, mood, human relation, and quality of life by stimulating the ‘fight and flight’ to maintain homeostasis (10). The response to stressors is important for a sense of well-being, productivity, and socialization. However, acute stress can become sustained in the presence of some negative socioeconomic factors such as financial problems, disease outbreaks (e.g. COVID-19 and Ebola outbreaks), job insecurity, loneliness, or bereavement. Negative socioeconomic factors could frustrate individuals and eventually activate the mechanisms responsible for chronic stress response (9).

Chronic stress may distort the normal metabolic, nervous, and immune responses (11), thus increasing the susceptibility to pathological conditions (12). Although stress has been clinically associated with male infertility, little is known about its possible effect on spermatogenesis and steroidogenesis compared with it inflammatory and oxidative stress mechanism. This narrative review provides compelling shreds of evidence, based on the available data from the literature, on the role of chronic stress on male fertility. It also provides information on the role of gonadotropin inhibiting hormone (GnIH) on male infertility.

## 2 GnIH

GnIH is a decapeptide hormone that plays a key role in the neural regulation of reproduction (13). It is one of the major hypothalamic neuropeptide hormones responsible for maintaining optimal reproductive functions (14). The novel hormone was discovered in the quail hypothalamo-hypophyseal system in the year 2000 and was named based on its inhibitory effect on the gonadotropic hormones and gonadotropic releasing hormone (GnRH) (15). This opened another research window in reproductive neuroendocrinology challenging the belief that GnRH is the only hypothalamic hormone responsible for regulating reproductive functions. Subsequently, the hormone was found to be present in most vertebrates including humans (16), and it has been established to exert influential activities on sexual behavior and gonadal functions (16).

**Figure 1:**
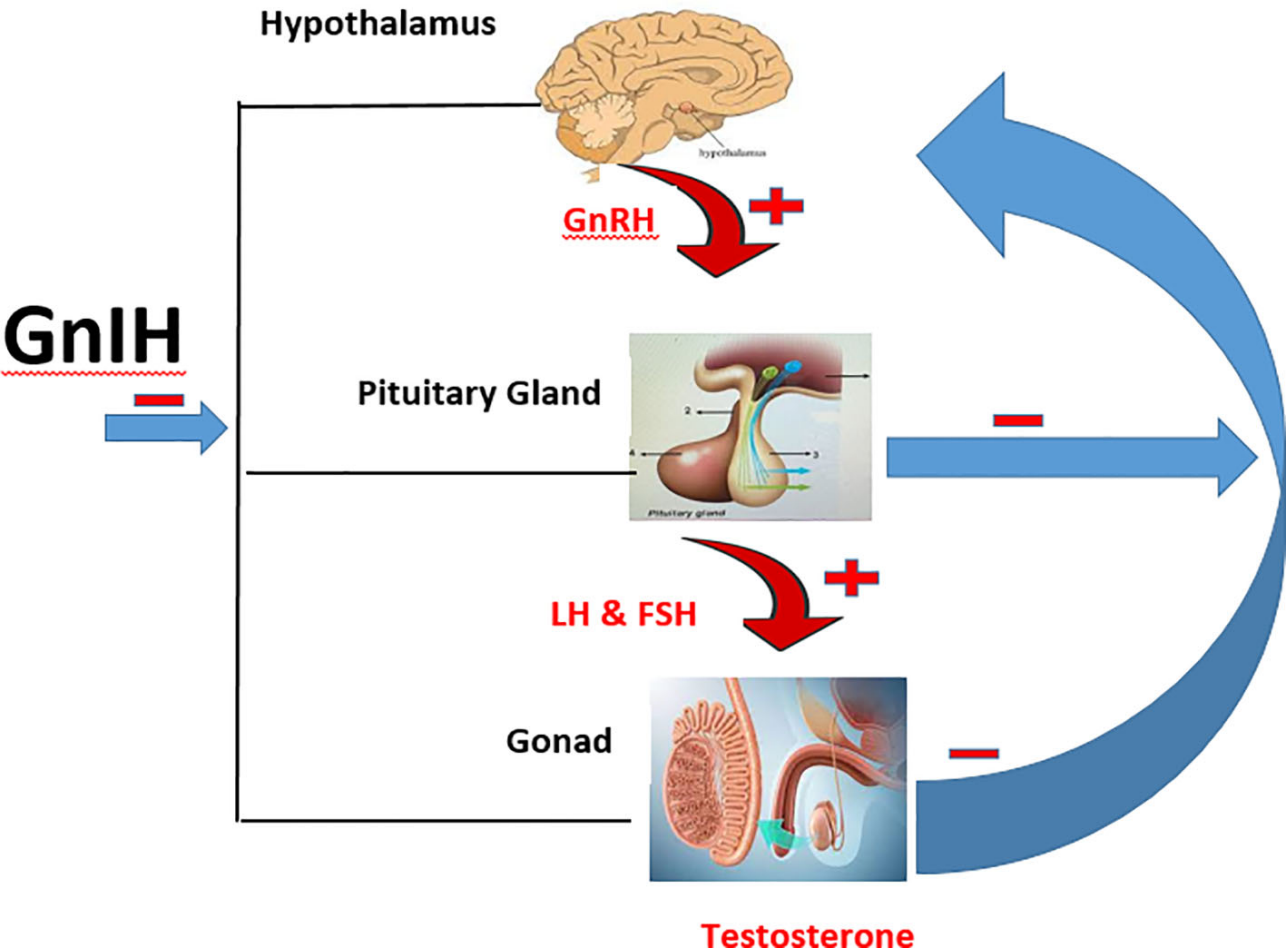
Schematic illustration of the HPG axis and the inhibitory effect of GnIH. ─ is inhibitory effect while ┼ is stimulatory effect.

GnIH is also referred to as RF amide-related peptides (RFRPs) in mammals, while it is known as LP*X*RF-amide in teleosts because it has LP*X*RF (*X* = L or Q) amide motif at the C-termini (14, 17). The two forms of GnIH found in mammals are RFRP-1 and RFRP-3, and have been identified to regulate the hypothalamic-pituitary gonadal (HPG) axis in men (13, 14, 18). GnIH directly inhibits GnRH since its axon is in contact with GnRH neurons (19, 20). Apart from its effect on the hypothalamus, it also inhibits the secretion of follicle stimulating hormone (FSH) and luteinizing hormone (LH) directly through its release into the hypothalamic–hypophyseal portal system (21). Furthermore, the presence of GnIH receptors on the testes of mammals is an indication that it may directly inhibit testicular functions (spermatogenesis and steroidogenesis). In fact, exogenous administration of GnIH has been shown to impair testicular development (22). Hence, GnIH can impair testicular functions at the level of the hypothalamus, pituitary, or testes (**Figure 1**).

Neurons responsible for the synthesis of GnIH are present in the mid-ventral continuum from the diagonal band of Broca to the mediobasal hypothalamus (23), while those regulating the secretion of gonadotrophic hormones extend to the median eminence to modulate the synthesis of FSH and LH (13, 24). The released LH and FSH then regulate the synthesis of gonadal hormones which in turn send negative feedback to the hypothalamus and anterior pituitary gland to keep the reproductive axis within the operating limits required for optimal reproductive functions (25). This closed loop is called the hypothalamic-pituitary-gonadal (HPG) axis, and it is known to be solely regulated in the hypothalamus via GnRH (26) until the discovery of GnIH.

### 2.1 Physiology of stress

Stress is an important phenomenon for survival that requires prompt physiological and behavioral responses for an individual to cope with the different situations in the environment and maintain body homeostasis (27). Physiologically, stress response can be categorized into the fast response mediated by the sympathoneural and sympathoadrenomedullary (SAM) axis and the slow response mediated by the hypothalamic-pituitary-adrenal axis (HPA) (28).

The sympathoneural and SAM are the first line of stress response, and their activation stimulates the release of catecholamine (epinephrine and norepinephrine) from the adrenal gland into the bloodstream. Also, norepinephrine becomes elevated in the brain due to its increased secretion and release from the sympathetic nerves (29). The released epinephrine and norepinephrine stimulate the alpha (α)- and beta (β)- adrenergic receptors found in the central nervous system, smooth muscles, and other organs of the body (30). The released epinephrine and norepinephrine bind to their specific membrane-bound G-protein receptors to activate the intracellular cyclic adenosine monophosphate (cAMP) signaling, which in turn stimulates various cellular responses (31). The activation of these receptors leads to an increase in heart rate, blood pressure, cardiac output, and skeletal muscle blood flow via vasoconstriction of both the smooth and cardiac muscles (32). They also stimulate an increase in blood glucose, lipolysis, oxygen utilization, and thermogenesis, and cause behavioral changes such as enhanced arousal, alertness, and attention (30).

In addition, the activation of the first line of the stress response stimulates the slow response, which is mediated by the activation of the HPA axis to release glucocorticoids (28). Corticotropin-releasing hormone (CRH) is secreted from the paraventricular nucleus (PVN) of the hypothalamus and binds to its receptors (CRH-R1 and CRH-R2). The CRH-R1 is the major receptor for activating the stress-induced secretion and release of adrenocorticotropic hormone (ACTH) (30). The release of CRH into the bloodstream stimulates the release of ACTH from the anterior pituitary gland into the bloodstream, which in turn stimulates the release of glucocorticoid from the adrenal cortex. HPA axis is regulated by the pituitary adenylate cyclase-activating polypeptide (PACAP) that regulates the release of CRH and modulates the HPA axis at different levels (33). PACAP also stimulates the secretion of catecholamines during autonomic response to stress (33).

The interplay between sympathoneural, SAM, and HPA axis in response to stress systematically produces metabolic and behavioral changes that are transient and adaptive (28). However, chronic sympathetic discharge and elevated levels of glucocorticoid during prolonged stress are associated with pathological conditions, such as metabolic disorder and infertility.

#### 2.1.1 Stress and infertility

Chronic stress is a psychological disorder that can lead to various sexual problems such as loss of libido and erectile and ejaculatory dysfunction (34). Besides, infertility itself is stressful because of its attendant complications such as social pressures, low self-esteem, unfulfilled desire, and a financial burden (35). Stress may impair testicular functions, which leads to reduced circulating testosterone, and impaired spermatogenesis and sperm quality (36). The first available report on the effect of stress on human spermatogenesis was obtained from death-sentenced prisoners kept for a long time before execution (37). The study reported impaired spermatogenesis that was so marked that the only cells found in the seminiferous tubules were the Sertoli and spermatogonial cells. In another study, milder stress was reported to significantly reduce circulating testosterone (37).

Various experimental results show a positive correlation between chronic stress and erectile dysfunction. Chronic stress impairs the normal morphology of the penile corpus cavernosum, which in turn impairs penile erection (34, 38). Additionally, chronic stress also `impairs endothelial function (39), which is important for penile erection via the No/cGMP signaling (40).

#### 2.1.2 Stress and GnIH

Chronic stress is positively related to the secretion of GnIH from the hypothalamus (41). In other to confirm the relationship between chronic stress and GnIH, adrenalectomy was performed in male rats, and it was observed that the increase in GnIH secretion under a stressful condition was abolished (42). Also, the study of Son et al. (43) revealed that glucocorticoid receptor (GR) is present in GnIH neurons that are located in the PVN, and treatment with glucocorticoids significantly increases GnIH secretion. GR is also expressed in rHypoE-23, which is a GnIH-expressing neuronal cell line from a rat hypothalamus (43). Stress-induced secretion and release of norepinephrine is responsible for stimulating the release of GnIH (44). Interestingly, the expression alpha-2A adrenergic receptors in GnIH neurons of male quail have been elucidated (44). Thus, it appears that the effect of chronic stress on fertility vis the HPA axis is mediated by the upregulation of the expression of GnIH.

Also, GnIH neurons and those of CRH are in direct contact with the PVN, and their release triggers the activation of the HPA axis (45). In addition, the CRH receptor is present in about 13% of the neurons of GnIH, and its activation upregulates GnIH-R *mRNA* (42). GnIH and cortisol are up-regulated in the presence of acute and chronic stress mediators (45). In agreement with the earlier findings of Kirby et al. (42), Higuchi et al. (46) demonstrated that GnIH and cortisol levels were markedly elevated during stress. However, like most neuroendocrine responses, sustained stimulation of the CRH receptor by chronic stress may lead to its desensitization, disrupting the HPA axis (47). Thus, it has been speculated that CRH-sensitive GnIH cells might also become desensitized by sustained chronic stress (48), thereby interrupting the GnIH-GnRH neuronal pathway. These pieces of experimental evidence show that CRH and GnIH are positively correlated since CRH can directly stimulate some parts of GnIH neurons, thereby increasing GnIH sensitivity via the upregulation of the GnIH-R.

Furthermore, chronic stress has been revealed to be associated with hypothyroidism by directly inhibiting the activities of the hypothalamic-pituitary-thyroidal (HPT) axis (49). Stress-induced secretion of glucocorticoids has been linked with hypothyroidism by inhibiting the conversion of thyroxine (T4) to triiodothyronine (T3). This is in agreement with the study of Kakucska et al. (50), which showed that the administration of dexamethasone and corticosterone led to a significant decrease in the expression of TRH *mRNA* within the hypothalamus (51). These observed stress-induced hypothyroidism might be accountable for the surge in GnIH following chronic stress. According to the study of Kiyohara et al. (52), transient hypothyroidism led to GnIH *mRNA* upregulation and delayed puberty onset in young female rats, these observed hypothyroidism-induced reproductive dysfunction was completely reversed in animals with genetic loss of GnIH. Similarly, Rodrigues et al. (53) reported that the regulatory effect of thyroid hormone on the HPG is via its effect on GnIH secretion. They reported that hypothyroidism is a potent stimulator for the release of GnIH which in turn decreased Kiss1 *mRNA* expression, and eventually inhibited the release of gonadotropins. This is in tandem with the study of Santos et al. (54) that also reported negative relationship between thyroid hormone and Kiss 1. Additionally, the presence of thyroid hormone receptors and response elements (52) further shows the direct relationship between thyroid hormones and GnIH. In fact, thyroid hormone regulates chromatin modifications of GnIH promoter to either stimulate or inhibit GnIH expression by H3acetylation and H3K9tri-methylation respectively (55). Hence, it is tempting to conclude that another possible mechanism underlying chronic stress-induced elevated GnIH could be due to its inhibitory effect on thyroidal function since hypothyroidism has been linked with elevated GnIH secretion and release.

Another possible mechanism of action responsible for stress-induced increase in GnIH secretion is via a leptin-dependent pathway. Chronic stress has been shown to increase leptin secretion (56), a peptide hormone that is produced and synthesized by the white adipose tissue (57). Leptin is a satiety hormone and has been established to play a role in reproduction by maintaining metabolism in the reproductive axis (58). Different animal studies have described the role of leptin in maintaining the HPG axis. The presence of about 15-20% of GnIH neurons on the long form of the leptin receptor (LepRb), suggests a possible relationship between GnIH and adiposity via leptin and feed intake (45). In fact, a significant decline in GnIH synthesis has been reported in the leptin-deficient ob/ob mice (59). This relationship could be traced to the pro-inflammatory activities of leptin. Increased circulatory leptin is positively related to the production of inflammatory cytokines and resistin (60). Leptin-induced inflammatory response could stimulate the secretion on GnIH, which in turn suppresses the HPG axis activities. This claim is supported by the study of Iwasa et al. (61) that reported the stimulatory effect of lipopolysaccharide on GnIH secretion. Hence, it is plausible that the observed increase in GnIH during chronic stress could be mediated via stress-induced increase in leptin secretion.

### 2.2 GnIH and HPG axis

The HPG axis is a crucial endocrine pathway that links the hypothalamus, pituitary, and gonads in the body (62). HPG is integral in the establishment and maintenance of normal physiological processes related to reproduction, such as sexual maturation, steroidogenesis, and spermatogenesis. It is responsible for the production of essential reproductive hormones, such as those involved in fertility and sexual maturation. Anatomically, The HPG axis is made up of the hypothalamus, that housed the KNDy and GnRH-producing neurons, the anterior pituitary, where the gonadotropes produced LH and FSH, and the gonads, responsible for sex steroids and gametes production.

The HPG axis is active in the human fetus till one year after birth, after which it goes quiescent till 10 years postnatal life when it becomes active again (63, 64). This is about the time for the onset of puberty. It is occasioned by an increase in GnRH secretion in a pulsatile way, leading to increased gonadotropin secretion as well (63, 64). Reduction in melatonin secretion due to the regression of the pineal gland, together with the increase in leptin and other hormones, contribute favorably to the reactivation of the HPG axis before puberty onset (65). Since melatonin is positively correlated with GnIH (66, 67), it can be speculated that a decline in the secretion of GnIH following melatonin reduction, has a role to play in the reactivation of the HPG axis during the pubertal stage.

The HPG axis is key in the regulation of reproductive functions in vertebrates (64, 65). While the two main hormones at the anterior pituitary level of the HPG axis, luteinizing hormone (LH) and follicle-stimulating hormone (FSH), stimulate gonadal functions (steroidogenesis and spermatogenesis) (68);, the release of these hormones is mainly regulated by neurons at the hypothalamic level that produces GnRH (15, 69). Pulsatile GnRH secretion can be triggered by environmental and tactile cues like food availability, photoperiod, rainfall, and the presence of a mate (70).

GnRH is a decapeptide hormone discovered to stimulate the release of LH and FSH from the pituitary gland of mammals (71). Early findings referred to GnRH as the Luteinizing Hormone-Releasing Hormone (LH-RH), until it was widely referred to as GnRH because of it stimulatory effect on not just LH, but also on FSH. While GnRH stimulates LH and FSH, its stimulatory effects on both secretions are not similar (72). Compared to LH, FSH secretion is more irregular in humans, which could be due to the pulsatility and different stimulatory effects of GnRH (73). This could also be a result of the existence of different gonadotropes subpopulations or different response times to GnRH (74). To support this claim, findings from an ovariectomized sheep administered GnRH antisera, revealed a complete inhibition of LH secretion (LH became undetectable within 24 hours), while FSH release fell slowly and remain detectable (75). Furthermore, the rate of GnRH input has been shown to selectively maintain the transcription of gonadotropin subunit gene. For example, rapid GnRH pulse rates upregulate α and LH-, while the slow pulse frequency increases FSH-β gene transcription (74, 76).

The gonadotroph cells located in the anterior pituitary are responsible for the production of LH and FSH. These cells are made up of large round cell bodies with pronounced Golgi apparatus and endoplasmic reticulum. These cells constitute about 10 to 15% of the functional anterior pituitary cell mass. The LH and FSH produced from these cells are from similar genes which accounted for their similar properties. LH and FSH are glycoproteins consisting of alpha and beta subunit. The alpha subunit is similar while the beta subunit of each hormone is different. The difference in the beta subunit gave each hormone its biological specificity. The alpha subunit of LH and FSH consist of 92 amino acids, while the LH beta sub unit is made up of 120 amino acids and FSH is made up of 118 amino acid (77). Additionally, LH consist of one-two sialic acid residues, which account for its shorter half-life, while FSH is madeup of 5 sialic acid residues, accounting for its longer half-life of 3-4 minutes. LH and FSH are responsible for maintaining gonadal functions. LH is majorly responsible for stimulating the Leydig cells to produce testosterone from the testis, while FSH is responsible for maintaining the Sertoli cells to maintain spermatogenesis. They both also maintain GnRH production via negative feedback mechanism.

Testosterone and its metabolite (dihydrotestosterone) are the androgen in the testis, and are the major male reproductive hormones in mature male mammals. Testosterone is required for maintaining spermatogenesis, and the production of mature sperm is intimately dependent on androgen action within the testis. In fact, the maintenance of optimal sexual and erectile function depends on the effective testosterone secretion (78, 79). Testosterone is also responsible for different biological processes and is important for the development and maintenance of male secondary characteristics. Testosterone is also responsible for maintaining the HPG axis via its negative feedback mechanism to the pituitary gland and hypothalamus.

GnIH is another hormone responsible for maintain the activities of the HPG axis. It is the first hypothalamic neuropeptide found to have an anti-gonadotrophic effect on all vertebrate species by directly inhibiting GnRH via the 2 G-ptotein coupled receptors - GPR147 and GPR74 which have been recognized as GnIH receptors (GnIH-R) of which GPR147 cDNA are found in the brain and pituitary while GPR74 cDNA are conveyed in some tissues. It is important to note that GPR147 is considered the principal GnIH receptors because of its higher binding affinity as compared to GPR74 (55). GnIH also acts by downregulating *mRNA* levels of luteinizing hormone beta-subunit (LHβ) and inhibiting its release from the anterior pituitary gland (16, 80). GnIH can also inhibit LH synthesis via its stimulatory effect on prolactin secretion (81), which is a potent inhibitor of LH secretion (82). Its action on follicle-stimulating hormone beta-subunit (FSHβ) is not clear since its studies in quail have shown no effect on *mRNA* levels or FSH release. Whereas, in cockerels and other avian species, LH and FSH were suppressed (83–85). In mammals, it is less evident and controversial, particularly in its correlation with puberty (83).

### 2.3 GnIH and kisspeptin

Apart from the direct inhibitory effect of GnIH on the HPG axis, it also inhibits the secretion of kisspeptin, which stimulates the release of GnRH from the hypothalamus. Kisspeptin is made up of 52-54 amino acid which cleaves from its precursors and amidation occurs in the C-terminals. While C-terminals 10 amino acids (Kp-10) are similar in mice, rats, cattle, sheep and pigs, in humans tyrosine in the C-terminals is substituted with phenylalanine (86).

Kisspeptin together with its receptor (GPR54/Kiss-1r) are responsible for controlling reproduction and puberty in mammals through their direct stimulatory effect on the GnRH neurons (87, 88). In fact, the gain and loss of function in KISS1/KISS1R genes mutations led to precocious puberty and hypogonadotropic hypogonadism respectively, in human and animal models (89). This is associated with the precocious stimulation or impairment of the HPG axis at the level of the hypothalamus. The presence of Kiss1R on GnRH-secreting neurons membrane further substantiate the direct relationship between kisspeptin and the hypothalamus (90). Aside the direct effect of kisspeptin on the HPG-axis at the hypothalamic level, kisspeptin also modulate the HPG-axis activities at the pituitary and gonadal level. This is supported by the fact that kisspeptin neurons are also intermediate in the sex-steroid mediated feedback mechanisms on reproduction (90). In fact, environmental cues such as environmental toxicants, stress, and diet interferes with HPG-axis activities via Kiss-secreting neurons consisting of kisspeptin/neurokinin B/dynorphin A (KNDy neurons) (89). KNDy neurons are proposed to form the long elusive GnRH/LH pulse generator (91). These neurons are responsible for modulating gonadotropin release and reproductive functions based on peripheral signals (92). In tandem with this claim, KNDy neurons have been shown to maintain reproductive and non-reproductive functions such as negative feedback control of gonadotropin release (93), metabolism (94), stress-induced cues on fertility (95);, and thermoregulation (96).

Although kisspeptin maintains reproductive functions via its modulatory effect on the HPG-axis, it’s effect on the peripheral organs cannot be overlooked. Kisspeptin system has been identified in the testis (97), suggesting its possible autocrine and paracrine intra-testicular communications activities, testosterone synthesis, and sperm production and quality. In fact, kisspeptin, but not GnRH has been identified in the plasma, and the amount was dependent on fertility status (98). Intriguingly, gonadotropin stimulation is not always sufficient in ameliorating the impaired steroidogenesis and spermatogenesis in clinical cases of KISS1R inactivating mutations (89, 99). Additionally, the specific reactivation of the Kiss1R gene in the GnRH secreting neuron of KISS1R−/− knockout mice does not successfully ameliorate the associated impaired spermatogenesis (100). These above pieces of information support our claim that testicular Kiss1R signaling is also important for maintaining steroidogenesis and spermatogenesis.

Based on the above importance of kisspeptin, it is plausible to infer that GnIH inhibit gonadotropin secretion via its inhibitory effect on kisspeptin. Coincidentally, the presence of GnIH-Rs in approximately 9-16% of RP3V kisspeptin neurons in rats (101), and 5- 10% of the anteroventral periventricular nucleus (AVPV) and 25% of ARC Kiss1 neurons in mice (102), further substantiate the direct relationship between GnIH and kisspeptin. Also, GnIH fibers and Kiss 1 neurons are closely located, suggesting that GnIH might be inhibiting reproduction via its direct inhibitory effect on kisspeptin neurons (52). Furthermore, GnIH-R and GPR54 knockout mice displayed a disrupted LH secretion; however, the disruption was prominent in GPR54 knockout mice. In addition, Kiss1 *mRNA* was observed to be unregulated in GnIH-R knockout mice, while about 33% increase in GnRH neurons was also observed (45, 103, 104). These pieces of information suggest multiple pathways for GnIH inhibitory effect on the HPG axis. Hence, GnIH can inhibit the HPG axis by inhibiting GnRH and/or kisspeptin neuron expression.

#### 2.3.1 GnIH and steroidogenesis

GnIH and its receptor have been reported to be expressed in the hypothalamus and gonads (105). This may infer that GnIH does not only inhibit the HPG axis at the level of the hypothalamus and pituitary gland, but also the level of the gonads (45). Aside from the presence of GnIH receptor in the gonads, GnIH *mRNA* transcripts have also been found to be synthesized in the testis and localized interstitium (106, 107). This reveals that GnIH may inhibit testosterone production by suppressing the HPG axis or eliciting a direct inhibitory effect on the testis. GnIH treatment has been found to significantly disrupt testicular functions by directly impairing testosterone production from the testis (107).

Testosterone production is a *de novo* synthesis that involves the transportation of cholesterol from the outer mitochondrial membrane to the inner part, which is a rate-limiting step in the biosynthesis of testosterone. This intra-mitochondrial transport is regulated by the steroidogenic acute regulatory (StAR) protein. In humans, the administration of GnIH down-regulated steroidogenic acute regulatory (StAR) activities, while the administration of GnIH antagonists up-regulated StAR activities (105). Outside steroidogenic enzymes, GnIH has also been shown to impair glucose homeostasis which stimulates the uptake of cholesterol, which is the precursor of steroid hormones (108, 109). Decline cholesterol and StAR activities may mediate GnIH-induced suppression of testosterone synthesis.

#### 2.3.2 GnIH and spermatogenesis

Spermatogenesis is the process of producing sperm cells from spermatogonial cells. This process starts during puberty and continues throughout a man’s life. Spermatogenesis is regulated by a complex interplay of hormones and signaling molecules, including GnIH. Recent findings have revealed that GnIH plays a negative role in spermatogenesis by disrupting the HPG axis (110), which is responsible for the control of gonadal function, including the regulation of spermatogenesis, sperm quality, and sperm function. RFRP-3 is considered to be similar to GnIH in terms of its effect on gonadotropin secretion in mammals and has been observed to exert both autocrine and paracrine action on the gonads where it directly inhibits testicular functions. Bentley et al. (106) reported that GnIH is synthesized in the seminiferous tubules and interstitial cells in birds, while McGuire et al. (111) reported that it inhibited testosterone synthesis in avian testicular cell culture. Bentley et al. (106) also identified the expression of GPR 174 - RFRP-3 receptors - in the epididymis, and vas deferens of birds. Rats (112), sheep (113), mice (105), Syrian hamsters (114), pigs (115), primates and humans (107, 111) can produce RFRP-3 in their gonads. Zhao et al. (114) and Ubuka and Tsutsui (116) also identified RFRP-3 and its receptors in spermatocytes and spermatids through immunohistochemistry and *in situ* hybridization and recorded an increase in the expression of RFRP-3 and GPR147 in late spermatocytes, signifying RFRP-3’s role in the maturation of sperm. Anjum et al. (117) studied the expression of GnIH in the testis of mice and correlated it with serum testosterone levels from birth to senescence and found that RFRP-3 may cause pubertal activation of senescence in mice testis.

In 118 and his team studied the impact of the GnIH homolog RFRP-3 on the production of sperm and steroids in mice and discovered that treatment with RFRP-3 caused a significant decrease in the levels of circulating steroids, and testicular activity in the mice. It also caused dose-dependent changes in spermatogenesis, such as a decrease in cell proliferation and survival markers, and an increase in markers of cell death in the testes. Both *in vivo* and *in vitro* studies showed that RFRP-3 had an inhibitory effect on testosterone production in the testes. RFRP-3 also suppressed the expression of the LHCGR receptor, StAR protein, and enzymes involved in steroid synthesis (CYP11A1 and 3β-hydroxysteroid dehydrogenase) in the testes, leading to dose-dependent suppression of testosterone secretion that is an important factor in spermatogenesis. Testosterone is required for processes that are critical for spermatogenesis including maintaining the BTB, supporting the completion of meiosis, the adhesion of elongated spermatids to Sertoli cells, and the release of sperm (119).

Sperm quality refers to the number, motility, viability, and morphology of sperm cells (78, 120, 121). Factors such as age, lifestyle, ejaculatory abstinence length, and genetic background can affect sperm quality (120). Recent findings have revealed that GnIH may alter sperm quality (122). Marques and Boguszewski (122) demonstrated that GnIH exposure reduces sperm count, motility, and morphology in an animal model. GnIH has also been shown to induce oxidative stress and inflammation, which may contribute to a decline in sperm quality (123).

In addition, GnIH has been reported to affect sperm function (112). GnIH and its regulation of the HPG axis have been associated with several pathologies and disorders related to reduce sperm function (124). GnIH has been found to impair capacitation and acrosome reactions (124). Also, studies have shown that GnIH reduces the fertilizing ability of sperm cells in animals (114, 116).

## 3 Conclusion and future perspective

In conclusion, stress causes GnIH-induced degeneration of testicular cells and impaired testicular and adrenal steroidogenesis, spermatogenesis, and sperm quality through the activation of the hypothalamic-pituitary-adrenal axis, inhibition of the hypothalamic-pituitary-thyroidal axis, leptin hypersecretion, and suppression of the hypothalamic-pituitary-testicular axis and kisspeptin release. More studies exploring the roles and associated mechanisms of GnIH in male infertility are recommended. Also, suppression of GnIH may likely be a beneficial preventive and therapeutic strategy to avert the negative effects of GnIH, especially during exposure to stress.

## Author contributions

AO: Conceptualization, Data curation, Formal analysis, Funding acquisition, Investigation, Methodology, Project administration, Resources, Software, Supervision, Validation, Visualization, Writing – original draft, Writing – review & editing. RA: Data curation, Formal analysis, Investigation, Methodology, Project administration, Resources, Software, Supervision, Validation, Visualization, Writing – review & editing. GB: Conceptualization, Investigation, Methodology, Project administration, Resources, Writing – review & editing. MH: Investigation, Methodology, Project administration, Resources, Writing – review & editing. LO: Data curation, Formal analysis, Funding acquisition, Investigation, Methodology, Project administration, Resources, Software, Supervision, Validation, Visualization, Writing – review & editing.
